# Fructose overconsumption-induced reprogramming of microglia metabolism and function

**DOI:** 10.3389/fimmu.2024.1375453

**Published:** 2024-03-26

**Authors:** Kenneth K. Y. Ting

**Affiliations:** ^1^ Department of Immunology, University of Toronto, Toronto, ON, Canada; ^2^ Toronto General Hospital Research Institute, University Health Network, Toronto, ON, Canada

**Keywords:** microglia, immunometabolism, macrophages, fructose metabolism, GLUT5, inflammation, glycolytic reprogramming, neurological dysfunction

## Abstract

The overconsumption of dietary fructose has been proposed as a major culprit for the rise of many metabolic diseases in recent years, yet the relationship between a high fructose diet and neurological dysfunction remains to be explored. Although fructose metabolism mainly takes place in the liver and intestine, recent studies have shown that a hyperglycemic condition could induce fructose metabolism in the brain. Notably, microglia, which are tissue-resident macrophages (Mφs) that confer innate immunity in the brain, also express fructose transporters (GLUT5) and are capable of utilizing fructose as a carbon fuel. Together, these studies suggest the possibility that a high fructose diet can regulate the activation and inflammatory response of microglia by metabolic reprogramming, thereby altering the susceptibility of developing neurological dysfunction. In this review, the recent advances in the understanding of microglia metabolism and how it supports its functions will be summarized. The results from both *in vivo* and *in vitro* studies that have investigated the mechanistic link between fructose-induced metabolic reprogramming of microglia and its function will then be reviewed. Finally, areas of controversies and their associated implications, as well as directions that warrant future research will be highlighted.

## 1 Introduction of microglia

Microglia, which are tissue resident macrophages (Mφs) of the central nervous system, constitute a major component of the innate immune system in the brain. Depending on the area, microglia can comprise 5% to 12% of the total cell populations in the brain (1). Although microglia are classified as tissue resident Mφs, their ontogeny is notably distinct when compared to other types of Mφs. Genetic fate-mapping studies have now demonstrated that microglia are derived from primitive Mφs, which are differentiated from erythro-myeloid precursors in the yolk sac, and they colonize the developing brain as early as embryonic day 9.5 (2–4). Upon colonization, they retain their embryonic origin throughout adult life with no replacement from circulating monocytes.

To provide immune protection and maintain a healthy neural microenvironment, microglia are responsible for monitoring and scavenging the parenchyma continuously through its interaction with environmental cues, such as chemokines, cytokines, and other trophic factors (5–7). Upon detection of changes in the local environment, depending on the type of stimuli, microglia becomes activated and display a diverse profile of phenotypes. Similar to other tissue resident Mφs, microglia can also be divided into M1 and M2 Mφs. As M1 Mφs, they display cytotoxic and pro-inflammatory responses upon recognition of inflammatory stimulus, such as bacterial lipopolysaccharides (LPS). On other hand, they can also be alternatively activated into M2 Mφs, where they can perform repair and regeneration functions (M2a), immune-regulatory functions (M2b) or the acquisition of a deactivating phenotype (M2c) (8, 9). Overall, microglia demonstrate high phenotypic and functional plasticity in response to the wide spectrum of changes that take place in the brain (1, 10). Apart from its scavenging functions, microglia are also now recognized to be involved in other dynamic processes that can modulate brain functions, including the formation and maturation of synapse, brain homeostasis, neurogenesis and regulating neuronal excitability (11).

## 2 Metabolic reprogramming of microglia

Recent advances in the immunometabolism field have shown that the acquisition of altered metabolic adaptations due to the rewiring of metabolic circuits, known as metabolic reprogramming, is critical for the activation of immune cells and their functions. To support the heterogenous phenotypes and functions of microglia as described above, they are capable of metabolizing a variety of carbon sources, such as glucose (12), fructose (13), free fatty acids (14), lactate (15), and ketone bodies (16), in which the type of fuel it metabolizes can modulate its ability to perform its effector functions. In general, microglia express GLUT3 and GLUT5 to facilitate the uptake of glucose and fructose respectively (17–19). However, under inflammatory conditions, microglia can undergo metabolic reprogramming by transcriptionally activating the expression of glycolytic genes, such as glucose transporters (GLUT1) and lactate transporters (MCT1), to increase the breakdown of glucose (glycolysis) (15, 20, 21). More importantly, it has been shown that LPS-induced activation of microglia cell lines increased the rate of glycolysis while decreased the rate of oxidative phosphorylation (OXPHOS) (22). This shift from OXPHOS to aerobic glycolysis is known as the Warburg effect, which was first observed in cancer cells by Otto Warburg (23). Mechanistically, it is believed that this metabolic shift is due to the activation of mechanistic target of rapamycin (mTOR) complex 1 and hypoxia-induced factor 1α (HIF-1α) (24–26), which directly transactivate the expression of glycolytic and inflammatory genes, along with the activation of HIF-1α co-activators.

**Figure 1:**
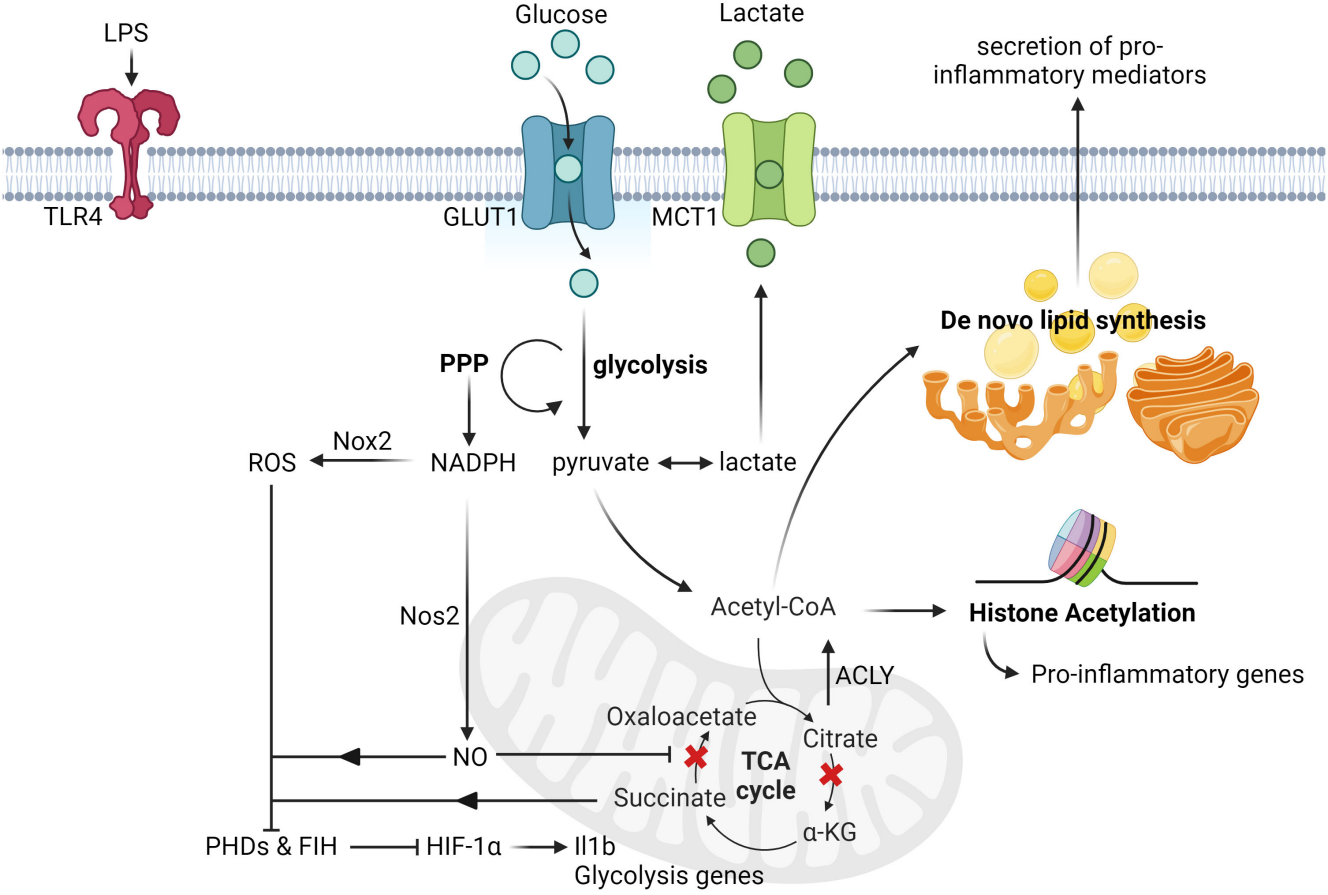
Glucose-induced metabolic reprogramming of activated microglia and macrophages. Upon LPS stimulation, microglia upregulate the expression of glucose transporters (GLUT1) and lactate transporters (MCT1) to increase the rate of glucose metabolism. This induction of glycolysis then leads to the increased rate of PPP, which is responsible for the synthesis of nucleotides and NADPH. The augmented production of NADPH subsequently drives the production of nitric oxide and ROS by Nos2 and Nox2 respectively. Upon nitric oxide-mediated inhibition of succinate dehydrogenase activity, it leads to the accumulation of succinate, which together with ROS, inhibit the activity of both FIH and PHDs. This eventually leads to the stabilization and activation of HIF-1α transactivation capacity, and the transcription of its targeted genes, such as *Il1b* and other glycolysis genes. Apart from this, another metabolic break happens at the isocitrate dehydrogenase level due to its suppressed expression post LPS stimulation, thereby leading to the accumulation of citrate. The rapid increased levels of citrate can then be converted to acetyl-CoA by ACLY and contribute to histone acetylation and *de novo* lipid biosynthesis (ex. synthesis of fatty acids), which can promote histone acetylation of pro-inflammatory genes and secretion of pro-inflammatory cytokines respectively. All figures are created with BioRender.com.

The increased flux of glycolysis in activated microglia is directly linked to its ability elicit an inflammatory response as blocking glycolysis with 2-deoxy-glucose impaired its production of pro-inflammatory cytokines, such as Tumor Necrosis Factor alpha (TNF-α) and Interleukin-6 (IL-6), in a NF-kB-dependent manner (27, 28). On the other hand, incubation of microglia with increased concentration of glucose further enhanced its production of TNF-α (29, 30). Although the direct link between the consequence of increased glycolytic flux and production of inflammatory cytokines is an ongoing investigation, recent reports have shown that the induction of glycolysis could lead to increased flux of the pentose phosphate pathway (PPP) and multiple disruptions in the tricarboxylic acid (TCA) cycle of Mφs in general (31–33). While the increased flux of PPP increased the production of NADPH, which is critical for regulating Mφ inflammatory responses (34), the disrupted TCA cycle had led to the accumulation of key metabolites that are linked to inflammation. For instance, the accumulation of succinate due to the inhibition of succinate dehydrogenase is important for the stabilization of HIF-1α and its direct transcription of *Il1b* transcripts (35, 36). On the other hand, the accumulation of citrate due to the inhibition of isocitrate dehydrogenase (36, 37) can be converted back to acetyl-CoA by ATP citrate lyase and it is critical for histone acetylation of inflammatory genes (38, 39), as well as *de novo* lipid synthesis, which supports the secretion of pro-inflammatory mediators (40). Apart from metabolites, the increased flux of the PPP also fuels the production of nitric oxide (NO) by nitric oxide synthase, in which the production of NO could inhibit the function of the electron transport chain (ETC) in Mφs (41, 42). The impaired activity of the ETC then induce the reversal of electron flow and drive the production of ROS, which is responsible to inhibit the activity of prolyl hydroxylases and thus stabilizing HIF-1α levels (43). More recently, it has also been revealed that blocking the activity of factor inhibiting HIF (FIH), an enzyme that inhibits the transactivation capacity of HIF-1α, is also critical for fully activating HIF-1α transcriptional function in Mφs (44). Collectively, these studies have shown that glucose metabolism plays a critical role in orchestrating the inflammatory response of activated microglia (**Figure 1**).

On the other hand, under anti-inflammatory conditions, such as stimulation by Interleukin-4 or Interleukin-13, microglia are metabolically adapted to utilize OXPHOS, which led to an attenuated uptake of glucose and production of lactate, and display similar oxygen consumption rate (OCR) and extracellular acidification rate (ECAR) levels as unstimulated microglia (14, 31). Finally, under homeostatic physiological conditions, microglia utilize a combination of oxidative catabolism of glucose and free fatty acids to fuel the TCA cycle and electron transport chain for generating large amounts of ATP, thereby sustaining the energetic needs for their basic surveyance functions (14, 45).

## 3 Fructose-induced metabolism in the brain and its reprogramming of microglia

It is well-established that glucose metabolism is vital to fuel the inflammatory functions of microglia, yet the role of fructose metabolism in modulating microglia inflammatory response is largely unexplored. In fact, it was previously unclear if dietary fructose consumption could even affect fructose metabolism in the brain as the breakdown of fructose mainly takes place in the intestine and liver (46–49). Until recently, Hwang et al. have used ^1^H magnetic resonance spectroscopy (MRS) scanning to measure intracerebral fructose levels, and found it was rapidly increased in the human brain under hyperglycemic condition (intravenous injection of 20% dextrose in human subjects) (50). Specifically, the authors found the fructose levels in the brain rose significantly, as early as 20 minutes post dextrose injection, and maintained elevated until the end of the study (240 minutes), with the range of fructose concentration changing from 0 to 0.7mmol/L approximately (50). More importantly, the authors also discovered that the rapid rise of intracerebral fructose levels occurred prior to the increase of plasma fructose levels, suggesting that the hyperglycemic condition could induce endogenous fructose production in the brain (50). Similar findings were also observed in a previous study performed by Hwang et al, where the authors have used gas chromatography-mass spectroscopy to measure fructose concentration in cerebrospinal fluids (CSF) from pregnant women and found that CSF-derived fructose and sorbitol levels were much higher (9-20-fold) than the levels in plasma (51). Taken together, these findings collectively demonstrate that fructose could be produced endogenously in the brain and that the effects of intracerebral fructose extend beyond its dietary consumption.

Understanding that fructose can be endogenously produced in the brain under hyperglycemic condition, and that a high-fructose diet is known to induce hyperglycemia, this then raises the possibility that a high-fructose diet can potentially modulate the metabolic and inflammatory profiles in the brain. Indeed, past reports have already revealed that a high fructose diet increased the expression of GLUT5 (52), as well as pro-inflammatory mediators, such as *Tnfa*, in the hippocampus (53). Mechanistically, fructose-diet induced Toll-like receptor 4/NF-kB and p38 MAPK/ERK inflammatory phosphorylating signaling cascades in the hippocampus, which eventually led to the increased expression of pro-inflammatory cytokines (54).

To further investigate if dietary fructose-induced inflammation in the hippocampus is due to increased activation of microglia, recent effort has been invested to elucidate the link between fructose-mediated metabolic reprogramming in microglia and its effector functions. Using leptin receptor-deficient type 2 diabetes mellitus (db/db) mice, Li et al reported an upregulation of fructose-related metabolism in the hippocampus, as determined by metabolomic, proteomic, and transcriptomic analysis (13). Single-cell RNA sequencing of hippocampus also revealed that the expression of fructose metabolism-related genes, such as *Khk* and *Slc2a5*, as well as ROS generating enzymes, such as NADPH Oxidase 4 (Nox4), were increased in microglia in db/db mice compared to littermate control (13). Mechanistically, selective knockdown of *Khk* in the hippocampus and microglia resulted in reduced expression of Nox4 in microglia (13). Fructose-mediated Nox4 regulation was also linked to its mitochondrial translocation and that Nox4-induced ROS impaired mitochondrial homeostasis, eventually leading to the damage of synaptic plasticity (13). Similar to this, another study done by Hyer et al also found that high fructose diet induced the activation of microglia in male, but interestingly not in female rats (55). Specifically, male rats fed on a high fructose diet had increased activation of microglia with reduction of their dendritic complexity, which correlated to an impairment of their reverse learning ability (55).

Although the studies above demonstrated that the metabolism and function of microglia *in vivo* was modulated in response to high fructose diet, *in vitro* studies that investigated the direct intrinsic effects of fructose on microglia remains to be controversial. For instance, Mizuno et al have treated primary microglia and SIM-A9 cells (murine microglia cell lines) with high concentration of fructose (7.5mM for 24h) and glucose (7.5mM for 24h) separately and found that only glucose has induced inflammation and expression of *Slc2a5* (GLUT5) (56). However, on the other hand, Xu et al have treated microglia BV-2 cells with high concentration of fructose (5mM for 24h) and found it induced TLR4/NF-κB activation, as well as pro-inflammatory gene expression, such as *Il1b*, *Il6* and *Tnfa* (57). Similar findings were also reported by Cigliano et al where they also incubated BV-2 cells with a range of concentration of fructose (0 to 10mM, 24h) and found it increased TNF-α production as measured by ELISA (54). Overall, these studies suggest that the intrinsic inflammatory effect of fructose appears to be dependent on specific experimental condition, and further experiments are needed to resolve the controversies.

Several important points of consideration need to be taken when comparing the results obtained from the presented *in vitro* and *in vivo* studies, one of which is the discrepancy between the intracerebral fructose levels quantified in the *in vivo* experiments, and the experimental fructose levels that were used in the *in vitro* experiments. For instance, as shown in the MRS scanning study performed by Hwang et al., the intracerebral fructose levels fluctuated only between 0 to 0.7mmol/L post dextrose injection (50). Yet, all the *in vitro* studies presented in this review have incubated microglia with fructose at a much higher concentration (at least 5mM). This not only raises concerns that the *in vitro* findings may not demonstrate physiological relevance, but also suggests the need of more *in vitro* studies that incubate microglia with physiologically relevant levels of fructose. Apart from this, the expression and function of proteins involved in fructose metabolism, such as GLUT5, is not always measured in the *in vitro* studies discussed in this review, thus making it difficult to draw a mechanistic link between the observed inflammatory phenotypes in microglia and their metabolism of fructose.

## 4 Conclusion and future perspectives

**Figure 2:**
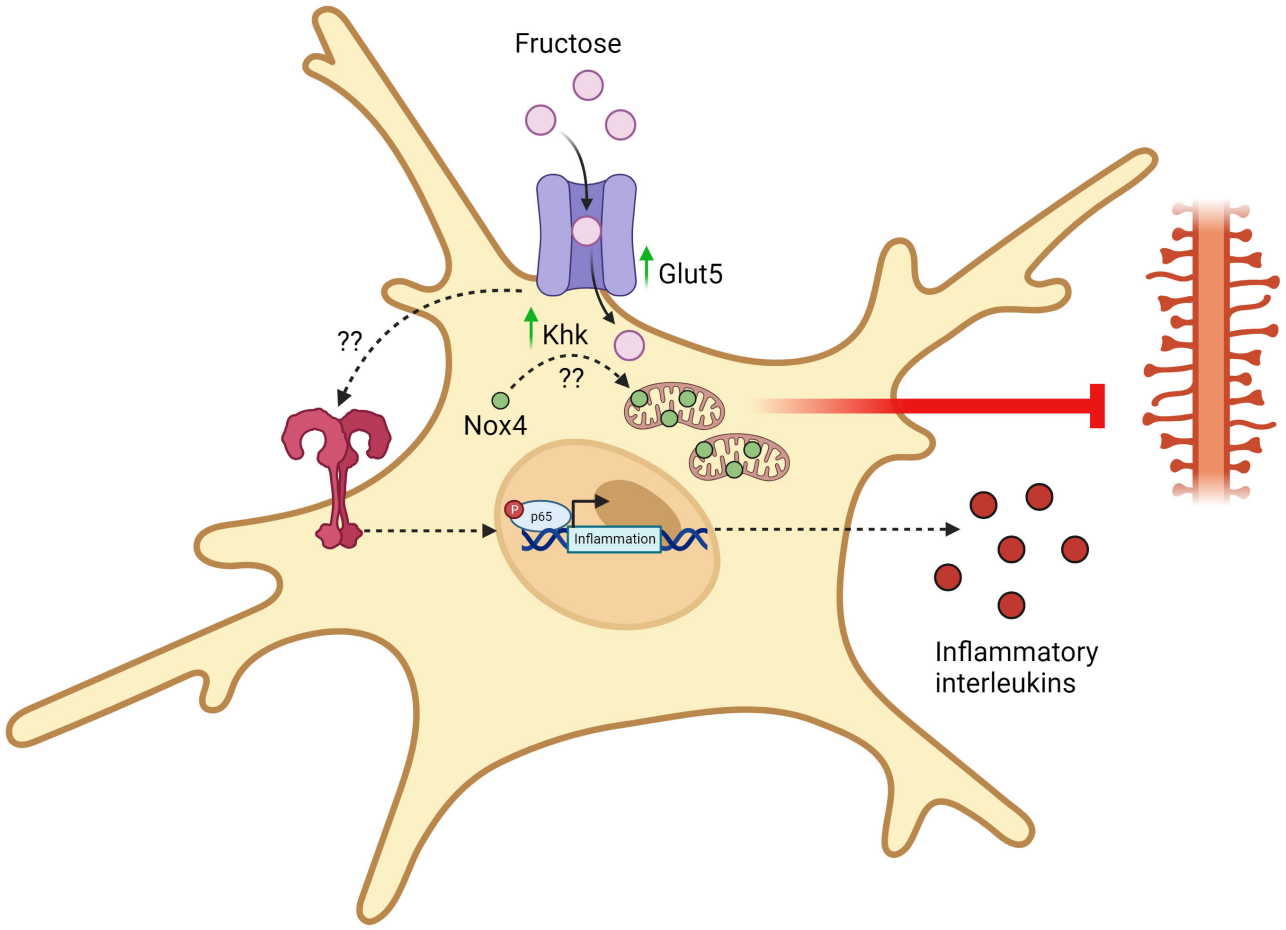
Fructose-induced metabolic reprogramming of activated microglia. Upon chronic exposure of high fructose, microglia upregulates the expression of fructose transporters (GLUT5) and keto-hexokinase (KHK). Through an unidentified mechanism, KHK promotes the translocation of Nox4 into the mitochondria and Nox4-mediated ROS synthesis disrupts mitochondrial homeostasis and eliminates dendrites. This eventually damages synaptic transmission and promotes the development of cognitive disorders. Apart from this, chronic exposure of high fructose also activates TLR4/NF-kB signaling pathways through an unidentified mechanism, thereby leading to the transcription and synthesis of pro-inflammatory mediators. All figures are created with BioRender.com.

The overconsumption of dietary fructose has been associated with the rise of chronic inflammatory diseases, such as obesity, diabetes, cardiovascular disease, and cancer (58–60). Furthermore, epidemiological studies have now revealed that high fructose consumption can also induce brain disturbances and negatively affect the development of neural system (61, 62). Early studies that investigated the relationship between the effects of dietary fructose and neural functions have shown that microglia do express fructose transporter (GLUT5) (18) and that fructose metabolism in the brain was stimulated under hyperglycemic condition (50). Understanding that the type of carbon fuel in which microglia metabolize can profoundly shape their effector functions, the focus of recent research has now shifted to the elucidation of a mechanistic link between high fructose consumption and its metabolic effects on microglia activation and inflammatory response. While this topic is still currently under heavy investigation, recent completed studies have generally demonstrated that high fructose-diet induced fructose metabolism in microglia is linked to its increased activation and inflammatory response, which can possibly lead to cognitive dysfunction and impairment (**Figure 2**). Yet, *in vitro* studies that directly investigate the intrinsic inflammatory response of fructose in microglia remains to be controversial and warrants future research to further determine the inflammatory signaling cascades that fructose metabolism may enhance, as well as the transcriptional regulation that modulates the expression of fructose metabolism genes.

The controversial findings observed *in vitro* could also suggest the existence of other external factors derived from the high fructose diet that could enhance the inflammatory responses of microglia *in vivo*, such as circulating metabolites that can cross the blood brain barrier. Furthermore, external variables such as sex can also modulate the effects of dietary fructose on neural functions. As described from the study by Hyer et al, cognitive flexibility was only impaired in male, but not female rats fed on high fructose diet, implicating that female sex hormones may play a role in the protection against fructose diets (55). Indeed, past research has shown that females do not develop hyperinsulinemia post fructose feeding with the exception of ovariectomy (63). Understanding the underlying differences between how high fructose consumption differentially affect the neural function of males and females may help to identify novel mechanisms that protect the deleterious effects of fructose in an *in vivo* setting.

## Author contributions

KKYT: Conceptualization, Funding acquisition, Visualization, Writing – original draft, Writing – review & editing.
